# Quantitative analysis of 3-dimensional facial soft tissue photographic images: technical methods and clinical application

**DOI:** 10.1186/s40510-015-0082-0

**Published:** 2015-07-02

**Authors:** Vikrum Nanda, Boris Gutman, Ehab Bar, Suha Alghamdi, Sotirios Tetradis, Aldons J Lusis, Eleazar Eskin, Won Moon

**Affiliations:** UCLA, California, USA; USC, California, USA; University of Connecticut, California, USA

## Abstract

**Background:**

The recent advent of 3D photography has created the potential for comprehensive facial evaluation. However, lack of practical true 3D analysis of the information collected from 3D images has been the factor limiting widespread utilization in orthodontics. Current evaluation of 3D facial soft tissue images relies on subjective visual evaluation and 2D distances to assess facial disharmony. The objectives of this project strive to map the surface and define boundaries of 3D facial soft tissue, modify mathematical functions to average multiple 3D facial images, and mathematically average 3D facial images allowing generation of color-coded surface deviation relative to a true average.

**Methods:**

Collaboration headed by UCLA Orthodontics with UCLA Neuroimaging was initiated to modify advanced brain mapping technology to accurately map the facial surface in 3D. 10 subjects were selected as a sample for development of the technical protocol. 3dMD photographic images were segmented, corrected using a series of topology correcting algorithms, and process to create close meshes. Shapes were mapped to a sphere using conformal and area preserving maps, and were then registered using a spherical patch mapping approach. Finally an average was created using 7-parameter procrustes alignment.

**Results:**

Size-standardized average facial images were generated for the sample population. A single patient was then superimposed on the average and color-coded displacement maps were generated to demonstrate the clinical applicability of this protocol. Further confirmation of the methods through 3D superimposition of the initial (T0) average to the 4 week (T4) average was completed and analyzed.

**Conclusions:**

The results of this investigation suggest that it is possible to average multiple facial images of highly variable topology. The immediate application of this research will be rapid and detailed diagnostic imaging analysis for orthodontic and surgical treatment planning. There is great potential for application to anthropometrics and genomics. This investigation resulted in establishment of a protocol for mapping the surface of the human face in three dimensions.

## Background

### Significance

The orthodontic profession is a specialty founded on the analysis and interpretation of geometries representing facial proportions. Dimensions and ratios are translated into representations of ideals and applied to facial aesthetics. The traditional foundation of orthodontics has relied on two-dimensional imaging to recreate the three-dimensional facial intricacies that exist in human anatomy. In the past decade, technological advancements have allowed visualization of bony anatomy and even more recently the soft tissues of the face. The orthodontic profession now has access to more information in three-dimensions than ever before [1]. It is our professional responsibility to utilize this information and apply these advancements in imaging towards patient care. There is a strong need for investigations which analyze progress and change in three dimensions to supplement and enhance traditional treatment modalities [2].

Although frontal and lateral cephalometric radiographs, panoramic radiographs, and intraoral and extra- oral photographs are still used, more emphasis has been placed on the 3D virtual image and soft-tissue esthetics [3, 4]. The paradigm shift in treatment philosophies also means that many clinicians have started to plan from the external profile, placing importance on the soft tissues of the face largely to determine the limitations of orthodontic treatment. From the perspectives of function, stability, and esthetics, the orthodontist must plan treatment within the patient’s limits of soft-tissue adaptation and contours [5].

Three-dimensional facial photographic imaging was introduced to orthodontics during the early years of the millennium. Research has demonstrated the value and increased accuracy of three-dimensional photography compared to traditional imaging modalities and has sought to develop new analyses for their application to clinical use [6, 7]. Most current methods for analysis of 3D photographic images involve simple visual evaluation or linear and angular measures between various point landmarks to evaluate facial aesthetics and proportions. The objective for this project is to develop and verify a protocol for mapping the surface of the human face in three dimensions using three-dimensional photography.

### Objectives and specific aims

Facial soft tissue analysis has evolved over time, and with the latest advancements in technology, 3D photographic imaging has created the potential for comprehensive facial evaluation. However, lack of practical true 3D analysis of the information collected from 3D photographic images has been the limiting factor holding back widespread utilization in the orthodontic profession. Current evaluation of these 3D facial soft tissue images relies largely on subjective visual evaluation and 2D point-to-point distances to assess aesthetics and facial disharmony. Those that have attempted 3D averaging and analysis have failed to comprehensively and accurately describe the 3D facial surface with respect to size, color, and morphologic difference.

Ultimately, this project will contribute to the creation of a true three-dimensional craniofacial analysis and establishment of a database of 3D photographic images for generation of age, gender, and race specific normative models of the human face. Overall, this study seeks to advance 3D imaging analysis technology by aiming to:

1. Apply and modify advanced technology used in brain mapping research to accurately and efficiently map the facial surface in 3D.
2. Apply and modify existing mathematical functions to find the average of multiple facial surfaces.
3. Develop protocol for superimposing sample faces on the averaged facial model, yielding a color-coded map of surface deviation and dysmorphology.
4. Superimpose and compare the average facial models of same patients before (T1) and after (T2) a particular treatment protocol (orthognathic surgery, MARPE/SARPE, RPE, etc.)

The immediate objectives of this project strive to map the surface and define boundaries of 3D facial soft tissue, modify and apply mathematical functions to average multiple 3D facial soft tissue photographic images, and mathematically average 3D facial soft tissue images allowing generation of color-coded surface deviation relative to a true average.

Accomplishing our immediate goal would result in a protocol for mapping the surface of the human face in three dimensions using 3D photographic images. Application of this technology would allow rapid soft tissue diagnostics for treatment planning in various health care specialties (i.e. orthodontics, oral/maxillofacial and plastic surgery. As this vision is realized, the ability to analyze patients or groups of patients in three dimensions would shift the diagnostic and normative paradigms currently used in craniofacial analysis towards an ever-progressive direction.

## Methods

### Quantification and averaging methods

The main purpose of this project is to create an average face from a random set of faces. We are interested in finding point-to-point correspondences across a large, potentially highly variable population of human face models. Using surface mapping, this project will refine and apply tools developed in neuroimaging research to create surface-based maps of the human face. On a broad level, the project can be divided into five parts, summarized below, and described in more detail in this section:

1. Collection of sample 3D Face models
2. Surface topology correction and spherical mapping
  - Non-manifold polygon correction
  - Boundary closure and Smoothing
  - Spherical Mapping
3. Shape registration
  - Initial Spherical Matching
  - Texture Matching
  - Geometry Matching and Registration Model
4. Average and distance map creation
  - Procrustes alignment/ Tensor Based Morphometry
  - Average and distance map creation, shape statistics analysis
5. Pilot study
  - Evaluation of individual shape morphology compared to averaged face

#### Collection of 3D face models

The novel nature of this investigation requires the formulation of a standard protocol for consistent image acquisition using the 3dMD facial imaging system. Our goal was to create an ideal environment and maintain consistent image acquisition for individual subjects over the duration of comprehensive treatment time. Natural head posture (NHP) was adopted for this study because it has been shown to be clinically reproducible [8–10]. IRB approval was acquired for this project.

#### Surface topology correction and spherical mapping

In non-technical terms, "topology" essentially refers to the number of handles, islands and boundaries of the surface. Since no well-defined correspondence between surfaces of different topologies is theoretically possible, one must perform a topological correction of each facial model before computing a dense correspondence. The simplest and most common approach is to make each surface topologically equivalent to a sphere.

##### Non-manifold polygon correction

Removing triangles (“faces”) and vertices of non-manifold nature from polygon models is a fairly common problem in 3D modeling.

##### Boundary closure and smoothing

We propose a boundary closure and surface extrapolation procedure similar to our previous work with shape correspondence [11]. Each boundary is initially “sewn together” with a new set of triangle faces, and the surface area of the new surface patch is minimized using standard linear optimization techniques with boundary conditions [12]. The triangulation of the patch is then subdivided into more faces, and the process is repeated iteratively, until the reduction in surface area is sufficiently small. This procedure is guaranteed to produce face models of spherical topology.

##### Spherical mapping

To enable efficient correspondence search across a dataset of faces, it is necessary to create an intermediate mapping to a common canonical space, where the final registration may be performed. A correspondence search on a sphere was performed, where all points moved around freely matching geometry appropriately, and matching extraneous tissue in some models to filler regions in others, as appropriate.

#### Shape registration

Several shape registration techniques exist for genus zero shapes (shapes of spherical topology), including those based on spherical parameterization. Among these are the rigid spherical cross-correlation [13], spherical demons [14], Laplace-Beltrami Eigen-function registration [15], just to name a few. The unique challenge for 3D face models, not addressed by existing methods, lies in the need to combine texture information from the coloring of the face and face geometry. Our proposed method would find dense correspondence across a set of faces using both texture and geometry information, while maintaining sufficient flexibility to deal with non-face regions of the model.

##### Initial spherical matching

To ensure a robust initial map, we used a curve-matching algorithm. A simple set of 10 curves was manually drawn on each face model using the BrainSuite 14 software, taking roughly 5 minutes per model by a trained operator. The correspondence between landmark points on the facial surface was determined automatically via the arclength map. We attain spherical displacements between corresponding curve points projected onto the sphere. This initial map suffices for further local refinement described below.

##### Texture matching

To minimize the mismatch between texture maps, we choose to use multi-channel Mutual Information criteria, often used to match 2D and 3D medical images from different modalities [16]. This choice is motivated by the fact that facial texture correspondence is characterized by complex relationships between intensities of different color channels, without a straight-forward transfer function. For example, different individuals may have entirely different color composition of their eyes and skin.

##### Geometry matching and registration model

In addition to texture, geometry mismatch will be simultaneously minimized following [14, 17] based on position- and orientation-invariant features such as mean and Gaussian curvature. We choose the fluid spherical registration model, which we developed recently in LONI’s past study in mapping the hippocampus in Alzheimer’s patients [17], because it is maximally flexible and completely agnostic with respect to the mismatch function, unlike previous methods. [13, 14] We believe that matching texture and geometry simultaneously will lead to the best most accurate mapping.

#### Average and distance map creation

##### Procrustes alignment/tensor-based morphometry

To compute the average face, we must first align the shape models in their original (not parametric) space, based on the computed dense correspondence. We will use the 7-parameter Procrustes method for this [18], excluding filler patches from the mismatch cost. An alternative measure of face morphometry, called Tensor Based Morphometry, has gained popularity in recent years [19]. Unlike distance-based features above, the TBM features invariant to the position and orientation of the shapes, making accurate Procrustes alignment a non-issue.

##### Distance and statistical maps

Having computed the average shape, we will compute the distance from the average to each shape at each point. Distance-to-average maps are displayed as colorized surface maps. Looking to the future, given a discrete, or continuous biological variable, such as whether the subject carries a certain gene, or some clinical measure of severity of a particular deformity, it is it possible to create statistical parametric maps based on distance to the average. These typically involve parametric or non-parametric (e.g. permutation) statistical tests done at each point [20], which localize the effect of the biological variable on the face surface.

Each single 3dMD individual image consists of roughly 32,000 vertices which represent an x,y,z coordinate on a Cartesian coordinate scale. Each averaged facial image retains this 32,000 vertex mesh with each single vertex possessing a specific variance. Therefore, superimposition of two average samples essentially represents a statistical p-map representing deviation from the norm.

#### Proof of methods/pilot study

A critical aspect in our analysis of the human face is to demonstrate that these methods can be used to average faces with variable morphology. In order to demonstrate our proof of methods, we have subjected our pipeline to several processes to demonstrate the function, accuracy, and potential applications for our pipeline.

A) A random non-homogenous sample of 10 subjects was selected with no exclusions on gender, age, and ethnicity. These subjects’ images were plugged into our averaging pipeline to create a true 3D average.
B) Furthermore, 3D distance‐to‐average maps displayed as colorized surface maps which will show individual deviation from our normative 10 sample average were created. This is a key feature that will allow for comparison of individual facial morphology to age, gender and race specific normative models for specific populations.
C) In order to demonstrate viability of the pipeline to average samples accurately over multiple time points, the T0 (initial) average of our 10 non-homogenous samples to their corresponding T4 (4 week) average was generated. This will illustrate the significance and accuracy of our average T0 to average T4 superimpositions and serve as a clear proof of methods.

## Results

### A) 3D facial photographic average

**Fig. 1:**
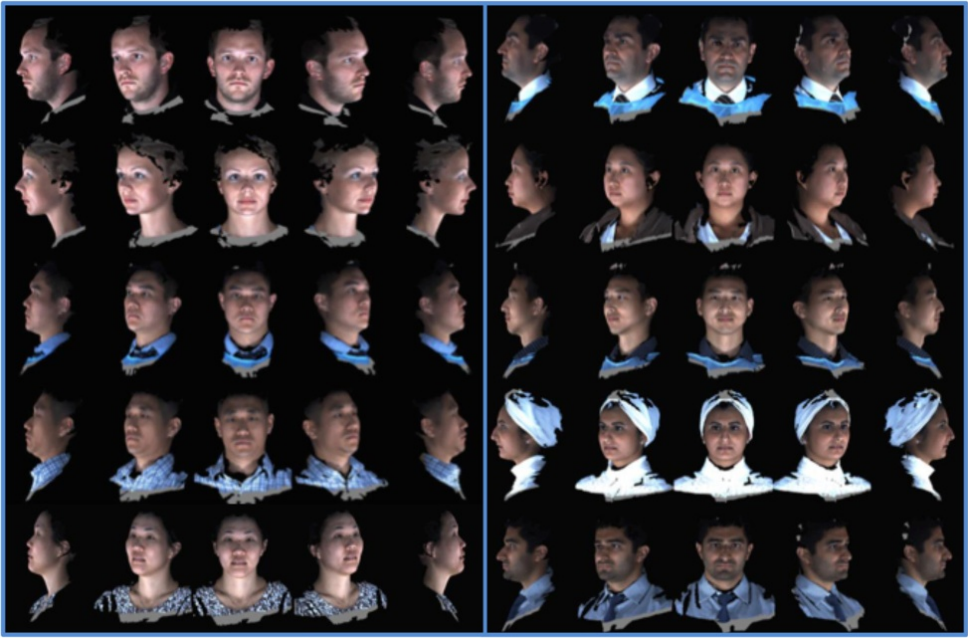
10 individual 3dMD samples used to create T0 average (Frontal, ¾, Lateral Views)

**Fig. 2:**
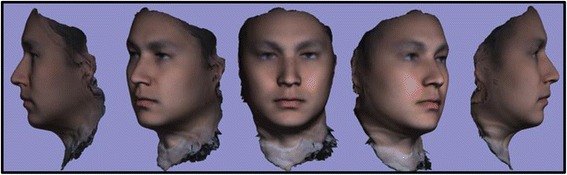
T0 average of 10 samples: “The Average Face”

A review of 3dMD clinical records resulted in a decision to use 10 3D photographic images to demonstrate proof of methods (Fig. 1). The sample population consisted of individuals with varying demographics, including ethnicity, gender, age, and skeletal/dental type. The purpose of using such a highly diverse sample was to demonstrate the power of these methods in generating a clean average model even with a population showing high topological variability. The 10 3dMD photographic images after topological correction were loaded into the initial software pipeline to generate the closed mesh forms of the images. These closed meshes were then traced for surface curves in areas of interest to aid in the “averaging pipeline”. The result of this dual pipeline process is a clean average of 10 human three-dimensional faces after topology correction, closed mesh creation, and shape registration (Fig. 2).

### B) 3D superimposition of individual sample to average

**Fig. 3:**
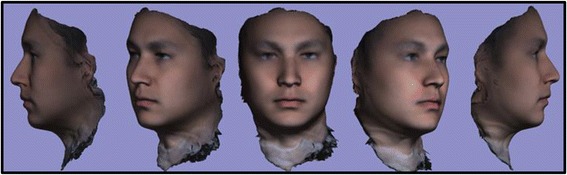
T0 average of 10 samples: “The Average Face” (Repeat of Fig. 2- Here for visual purposes)

**Fig. 4:**
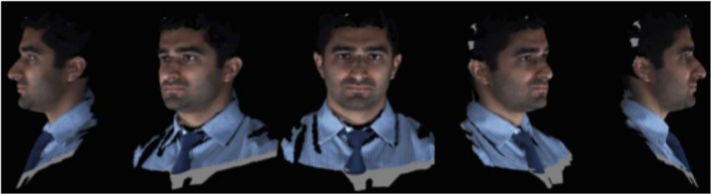
T0 3dMD image of individual sample to be compared to the average T0 norm above

**Fig. 5:**
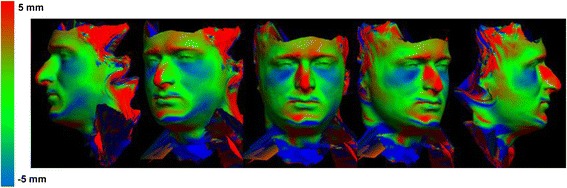
T0 individual sample superimposed over our T0 10 subject average norm. Scale is -5 mm to 5 mm

After development of an average facial normative model from the 10 image sample over two time points, our registration technique was applied to demonstrate the ability to superimpose a sample individual 3D facial image on the average model (Figs. 3, 4, 5). The orientation was maintained and registration was made possible thorugh the mesh framework, which retains point-to-point correspondence of vertices between shapes. The next step in producing clinically applicable superimpositions is to develop color-coded maps of surface deviation and dysmorphology. The color schematic that we chose to represent displacement values were as follows: Green represents no deviation from the norm; Blue represents deviation in a negative/inwards direction; Red represents deviation in a positive/outward direction. The magnitude of displacement ranged from -5 mm to 5 mm. Users have the ability to zoom in/out and manipulate the 3D superimposition to achieve any orientation desired to more appropriately view the results.

### C) 3D superimposition of 10 subject average T0 to 10 subject average T4

**Fig. 6:**
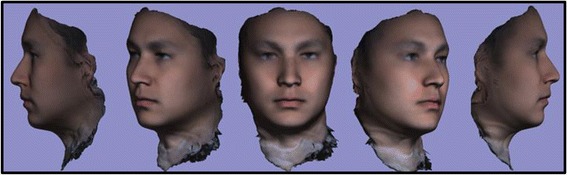
T0 average of 10 samples: “The Average Face” (Repeat of Figs. 2,3)

**Fig. 7:**
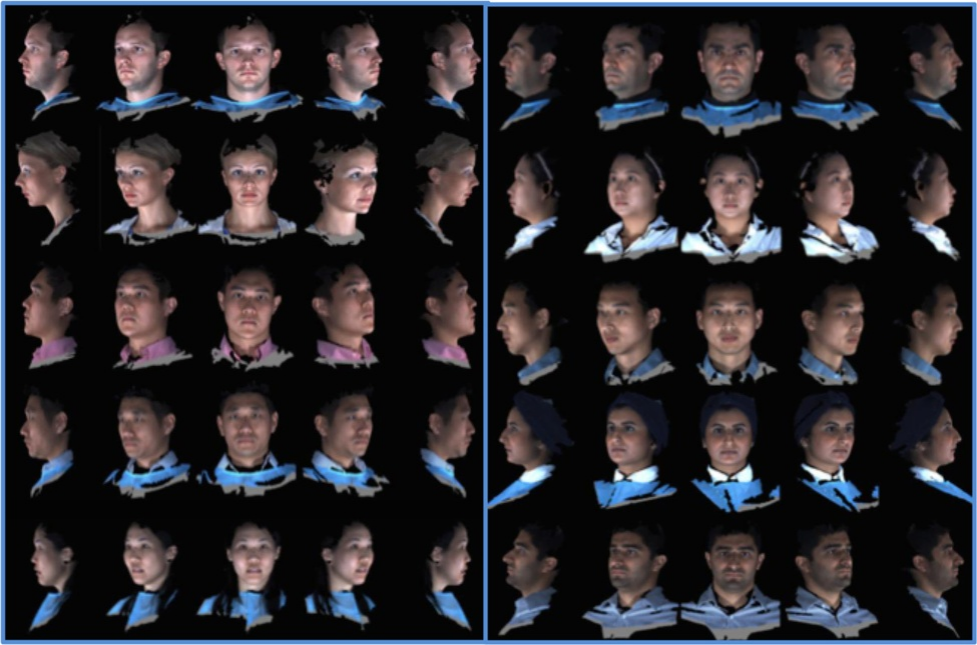
10 individual 3dMD samples used to create T4 average (Frontal, ¾, Lateral Views)

**Fig. 8:**
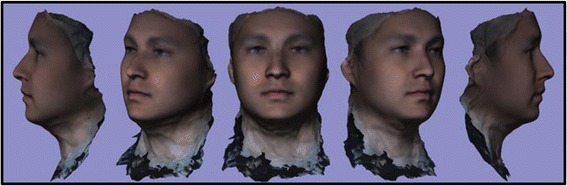
T4 (4 week) average of 10 samples: “The Average Face”

**Fig. 9:**
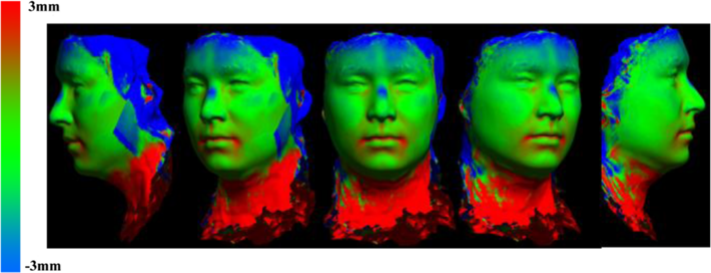
T0 average to T4 average 3D superimposition

A critical application of this averaging pipeline is to generate comparisons among groups of subjects. This goes one step beyond individual to average comparisons as seen in the previous section. Illustrating the ability to compare norm to norm over differing time points we have generated a 3D superimposition of our T0 10 subject average (Figs. 2, 3, 6) over our T4 10 subject average (Fig. 8). The scale has been adjusted for this superimposition to detect more subtle change among average to average comparison. For our purposes, the T0 average and T4 average should not vary significantly due to the fact that they were taken on subjects not undergoing any treatment modalities nor experiencing identifyable physiologic change over the 4 week test period. The scale used for the below results was -3 mm to 3 mm. An identical color schematic to the previous colorized displacement map was used here (Figs. 6, 7, 8, 9).

## Discussion

### A) Clinical applications

The overarching goal of this project was to successfully and accurately average the soft tissues of the human face in three dimensions. 3dMD 3D facial photographs were taken of subjects over two time points. The 3D images were piped into our developed software to process the data into a closed mesh format. The mesh geometries underlying the 3D facial photographs were topologically corrected to create a more continuous three dimensional mesh structure. Surface curves were traced using Brainsuite 14a software to identify areas of interest for the registration process. The geometries were then mapped to a sphere and aligned/registered to create an overall multi-subject average.

Creation of a true three dimensional average of the human face opens the doors to a variety of significant and novel applications. An immediate application of the basic average of a specific inclusion of samples may yield normative data for a specific demographic. This could be applied to anthropologic and genetic sciences. The ability to compare an individual to a generated average yields further potential to compare individual subjects against groups to determine detailed deviation and dysmorphology. This would prove to be invaluable in medical/dental specialties to determine appropriate treatment for correction of facial deviation from the norm.

One can imagine that comparison of norms at different time points could prove quite useful and informative when dealing with various aspects of longitudinal change. Examples include but are not limited to orthodontic treatment, plastic/ maxillofacial surgical outcomes, longitudinal change with aging, and data collection for surgical simulations. Comparisons using this technology can be made on a macro scale involving large populations and ethnicities. Acquisition of this data poses no risk to the patient of any kind and given one 3D photographic imaging machine allows acquisition of unlimited images relying only on digital storage capacity.

The impact to orthodontics will be speedy quantitative 3D comparison of patients relative to their 3-dimensional norms in diagnostics and treatment planning. Various research opportunities will arise as a direct result of our project as 3-dimensional normative soft tissue data using our pipeline can be readily generated for ethnic, race, and gender specific populations.

The immediate objective leading to possible future publication seeks to demonstrate our project’s impact to clinical orthodontics. We will follow patients over pre-treatment and post-treatment time points. We will generate a norm for the samples and create a colorized displacement map comparing the average norm pre-treatment in contrast with post-treatment. The methodologies described previously will intake individual 3-dimensional photographic patient records and output a generated average with quantifiable data allowing visualization and verification of treatment outcomes in the orthodontic profession.

### B) Conclusion and future directions

As completion of this project is realized, we will set our sights towards correlation of the hard and soft tissues of the craniofacial complex. Our anticipated objectives following this project involve:

1. Overlay of facial soft tissue maintaining color and texture data on their corresponding skull CBCT data
2. Generate functions to allow comprehensive averaging of multiple skulls with their corresponding facial soft tissue
3. Create accurate methods to quantify and demonstrate differences in the craniofacial complex of an individual compared to a norm
4. Allow accurate manipulation of combined hard and soft tissue essentially creating the most accurate virtual patient known to our profession

This project will allow for the revisions of existing 2-dimensional norms which are currently being used to educate the future orthodontists of our generation.

We believe that the strength of our investigation is in the application to our profession. A software pipeline generated to create true and accurate 3D averages of facial soft tissue will attract interest from orthodontic software corporations. However, the likely impact of our methods may transcend orthodontics. Physical anthropologists and fields of surgery (such as Plastics and OMFS) would have interest in the information of norms and surface deviations of individuals which would carry applications to their respective fields.
